# Feasibility, Acceptability, and Preliminary Outcomes of Participatory Contraceptive and Sexual and Reproductive Health Stigma-Reduction Workshops for Adolescent Girls and Young Women in Tanzania: A Mixed-Methods Pilot Study

**DOI:** 10.1177/15248399261483600

**Published:** 2026-09-11

**Authors:** Rachel Di Iorio, Nicola West, Anisia E. Kiwelu, Doreen A. Mruma, Elizabeth G. Ngolle, Mwasiti K. Jumanne, Isabell Kearn, Carmen H. Logie, Karen Yeates

**Affiliations:** 1 Queen’s University, Kingston, Ontario, Canada; 2 WEMA Health Innovation Tanzania, Moshi, Tanzania; 3 McMaster University, Hamilton, Ontario, Canada; 4 University of Toronto, Ontario, Canada; 5 United Nations University Institute for Water, Environment and Health, Richmond Hill, Ontario, Canada; 6 Women’s College Hospital, Toronto, Ontario, Canada

**Keywords:** child/adolescent health, global health, health promotion, reproductive health/reproductive justice, sexual health, stigma

## Abstract

Adolescent girls and young women in Tanzania experience stigma related to sexual and reproductive health (SRH) and contraceptive use, which can discourage care-seeking and constrain reproductive autonomy. This mixed-methods pilot study assessed the feasibility, acceptability, and preliminary outcomes of participatory stigma-reduction workshops delivered in urban and rural Kilimanjaro, Tanzania. Data were collected between February 7 and March 7, 2025. Forty adolescent girls and young women aged 15 to 20 participated in a 3-day workshop series facilitated by trained local female facilitators. The workshops were adapted from an established stigma-reduction curriculum and used participatory dialogue, critical reflection on gender norms, and peer learning. Participants completed pre- and post-workshop surveys assessing contraceptive use stigma, broader adolescent SRH stigma, and sexual double standards. Focus group discussions with participants and facilitators explored workshop experiences, acceptability, and perceived impacts. All participants completed the workshops and surveys, and qualitative findings indicated that the participatory format was engaging and acceptable. Contraceptive Use Stigma scores decreased from pre- to post-workshop (*p* < .001; Cohen’s *d* = −0.76), whereas changes in broader adolescent SRH stigma and sexual double standards were not statistically significant. Qualitative findings suggested increased comfort discussing SRH, reduced moral judgment toward contraceptive use, and greater confidence communicating with peers and adults, while community- and provider-level barriers persisted. These pilot findings support the feasibility and acceptability of participatory stigma-reduction workshops and justify longer-term evaluation using a controlled design.

## Introduction

Adolescents in Tanzania face high rates of early pregnancy and unmet need for contraception, despite the formal availability of sexual and reproductive health (SRH) services in public health facilities (Tanzania Ministry of Health & ICF, 2022). Social stigma surrounding adolescent sexuality and contraceptive use remains a critical barrier to care-seeking, particularly for adolescent girls and young women (AGYW). Fear of moral judgment by parents, health care providers, religious leaders, and community members often deters adolescents from accessing information and services, even when they recognize the potential benefits of contraception (Carroll & Kapilashrami, 2020; Chelva et al., 2022; Nyblade et al., 2017; Sedekia et al., 2017).

Stigma functions as a social and structural determinant of health, influencing not only individual attitudes but also adolescents’ access to supportive environments, credible information, and nonjudgmental care (Hall et al., 2018; Logie et al., 2019, 2024). In Tanzania and similar contexts, stigma related to adolescent SRH is closely intertwined with gender norms that prioritize female sexual restraint, obedience, and modesty while affording boys greater sexual freedom (Hall et al., 2018; Logie et al., 2019, 2024; Mushy et al., 2020). These norms contribute to unequal expectations, shame, and social surveillance that disproportionately constrain girls’ autonomy and decision-making. These gendered expectations are reflected in sexual double standards, whereby similar sexual behaviors may be judged differently for girls and boys. Girls may be stigmatized for sexual activity or contraceptive use, while comparable behaviors among boys may be viewed as more acceptable or expected. Because these double standards can reinforce contraceptive and broader SRH stigma, examining them alongside stigma-related outcomes provides insight into whether the workshops influenced the wider gender norms surrounding adolescent sexuality (Moreau et al., 2019, 2021).

Existing adolescent SRH interventions in low- and middle-income countries have primarily focused on improving knowledge, strengthening life skills, expanding youth-friendly services, or increasing access to information through schools, clinics, and digital platforms (Chandra-Mouli et al., 2014; Hartog et al., 2020; L’Engle et al., 2013). Although these approaches can address informational and service-access barriers, fewer interventions have directly addressed the stigmatizing social norms and moral judgments that shape adolescents’ willingness and ability to seek SRH information and services.

The broader stigma-reduction literature in low- and middle-income countries demonstrates that participatory education, facilitated reflection, social contact, and opportunities to challenge stigmatizing language can improve stigma-related knowledge and attitudes among adolescents and other populations (Hartog et al., 2020). Participatory stigma-reduction curricula have also been implemented in health facilities in Tanzania and other sub-Saharan African settings, particularly to address HIV- and drug-use-related stigma among health workers (Clay et al., 2024; Mlunde et al., 2024). However, these interventions have generally focused on other health-related stigmas or institutional settings rather than contraceptive stigma among AGYW in community settings. To our knowledge, no published intervention has specifically evaluated a participatory contraceptive stigma-reduction program for AGYW in Tanzania. This represents an important gap in adolescent SRH research and practice.

To address this gap, we adapted a participatory stigma-reduction curriculum, originally developed for health workers, for use with AGYW in community settings in Tanzania (Clay et al., 2024; Mlunde et al., 2024). The London School of Hygiene and Tropical Medicine’s Expanded Total Facility Approach curriculum was previously applied to HIV-related stigma in health facilities in Tanzania, Zambia, and Ghana and uses participatory activities to address health stigma.

This study was guided by the Health Stigma and Discrimination Framework, which conceptualizes stigma as a multilevel process involving drivers and facilitators, stigma marking, and manifestations that influence health outcomes across individual, interpersonal, institutional, and structural levels (Stangl et al., 2019). The framework informed the intervention’s attention to multiple sources and manifestations of stigma, including adolescents’ attitudes, peer and family expectations, community judgment, and experiences within health care settings. It also informed the selection of outcomes assessing contraceptive use stigma, broader adolescent SRH stigma, and related gender norms.

The Stigma Power Process Model complemented this framework by explaining how stigma can function as a form of social control that maintains unequal (Link & Phelan, 2014). Applied to adolescent SRH, this model helped situate moral judgments about girls’ sexuality and contraceptive use within gendered expectations that regulate girls’ behavior and constrain reproductive autonomy. It informed workshop activities that encouraged participants to identify, critically examine, and challenge the language, stereotypes, and gendered expectations through which stigma is reproduced.

Together, the frameworks provided complementary descriptive and explanatory functions. The Health Stigma and Discrimination Framework identified where and through what processes stigma operates, whereas the Stigma Power Process Model helped explain why stigmatizing norms are produced and maintained. Participatory and empowerment-oriented health promotion approaches were used to translate these theoretical perspectives into practice through dialogue, critical reflection, and peer learning (Wallerstein & Bernstein, 1988). Accordingly, adolescents were positioned as active contributors to knowledge construction rather than passive recipients of information. These complementary perspectives informed the workshop design, outcome selection, and interpretation of both changes in individual attitudes and the persistence of broader interpersonal and structural barriers.

The intervention was adapted in partnership with a Tanzanian community-based organization with long-standing experience in adolescent health promotion and gender-focused programming. Local collaborators contributed contextual knowledge regarding adolescents’ SRH experiences, locally salient forms of stigmatizing language, gendered expectations, and appropriate approaches to workshop delivery. Their involvement supported the linguistic and cultural adaptation of activities, scenarios, and facilitation strategies and strengthened the intervention’s relevance to adolescents in the participating communities.

## Purpose and Aims

The purpose of this study was to assess the feasibility, acceptability, and exploratory outcomes of participatory contraceptive stigma-reduction workshops delivered to AGYW in urban and rural Kilimanjaro, Tanzania.

The pilot focused on three domains: (a) participants’ knowledge, attitudes, and lived experiences related to contraception and SRH; (b) the feasibility and acceptability of the workshop program; and (c) preliminary changes in contraceptive stigma, broader adolescent SRH stigma, and sexual double standards. The term *pilot* refers to the study’s primary purpose of assessing feasibility, acceptability, and procedures to inform future evaluation, whereas *exploratory* refers to the preliminary pre–post outcome analyses, which were not designed to establish intervention efficacy.

## Methods

### Study Design and Setting

We conducted a mixed-methods, pre–post pilot study in one urban and one rural district in the Kilimanjaro Region of northern Tanzania. The urban setting has comparatively greater population density and geographic proximity to health facilities, pharmacies, schools, and other SRH information and service resources. In contrast, the rural setting includes more geographically dispersed communities, where distance, transportation, and more limited availability of adolescent-responsive services may affect access to SRH information and care. These settings were included to examine the feasibility and relevance of the intervention across differing social and service-access contexts.

The study was implemented in collaboration with a local community-based non-profit organization, with long-standing experience in adolescent health promotion and gender-focused programming. Organization staff contributed to intervention adaptation, recruitment planning, facilitation, and interpretation of the findings. Workshops were held in a community venue selected to provide a neutral, non-clinical space that was familiar and accessible to participants.

The study design prioritized feasibility, acceptability, and learning for practice rather than hypothesis testing (Thabane et al., 2010). The mixed-methods approach allowed for integration of quantitative measures of stigma-related attitudes with qualitative insights into participant experiences, engagement, and perceived impacts.

### Participants and Recruitment

Participants were AGYW aged 15 to 20 years residing in the study catchment areas. Recruitment was conducted through community networks, referrals from local partners, and snowball referrals. Eligibility criteria included age, residence in the study area, and willingness to participate in group-based workshops over 3 consecutive days.

A total of 40 AGYW participated, with workshops conducted separately in urban and rural sites and participants evenly distributed across locations. Four female facilitators with prior experience working with adolescents were recruited from the local non-profit organization and trained to deliver the intervention. Facilitators were selected to reflect participants’ linguistic and cultural contexts, an important consideration for creating trust and minimizing power differentials.

### Intervention Adaptation

The intervention was adapted from four participatory activities included in the London School of Hygiene and Tropical Medicine’s Expanded Total Facility Approach stigma-reduction curriculum, originally developed for health workers (Clay et al., 2024; Mlunde et al., 2024). Activities were selected based on their potential relevance to adolescent contraceptive and SRH stigma, including attention to stigmatizing language, personal experiences of stigma, gender expectations, and strategies for challenging stigma.

Adaptation was conducted in collaboration with staff from the Tanzanian community-based partner organization. The health-worker and facility-oriented framing of the original curriculum was replaced with adolescent- and community-focused content. References to HIV-related and facility-based stigma were adapted to address judgments surrounding adolescent sexuality, contraceptive use, gender norms, and access to SRH services. Activities were translated into Kiswahili and revised to incorporate locally familiar language, names, examples, and scenarios. Prompts were also adapted to reflect locally salient concerns, including moral judgments surrounding girls’ sexuality, expectations of female respectability and sexual restraint, and anticipated judgment from family members, community members, and health care providers.

The core participatory structure and stigma-reduction mechanisms of the original activities were retained, including facilitated dialogue, critical reflection, visual mapping, role-play, and collective problem-solving. Adolescents did not participate in a separate formal pre-implementation pilot-testing phase. Rather, the present study served as the initial pilot of the adapted intervention. Participants contributed feedback during workshop discussions and follow-up focus groups, which was used to identify areas for future refinement, including additional visual and practical contraceptive content and greater attention to mental health and community engagement.

### Intervention Delivery

The intervention consisted of four participatory activities delivered over 3 consecutive days in separate urban and rural workshop groups (Table 1). Sessions were conducted in Kiswahili by trained local female facilitators. Activities used group discussion, drawing and visual mapping, guided reflection, and role-play rather than didactic instruction. Facilitators used a structured workshop guide that specified each activity’s purpose, sequence, discussion prompts, and concluding messages while allowing them to respond to participant questions and group dynamics. The workshops emphasized voluntary participation, respectful dialogue, confidentiality within the group, and participants’ freedom not to disclose personal experiences.

**Table 1.** Participatory Stigma-Reduction Workshop Activities Delivered to Adolescent Girls and Young Women in Urban and Rural Kilimanjaro, Tanzania, February 2025.

Core activity	Estimated duration	Behavioral objectives	Theoretical constructs addressed	Activity and facilitator guidance
Outside the Gender Box	90 min	Participants identify community expectations associated with femininity and masculinity; examine how these expectations regulate girls’ sexuality and contraceptive decision-making; and identify examples of challenging restrictive gender norms	Gender norms; sexual double standards; stigma drivers and facilitators; gendered power and social control; critical reflection	Participants collaboratively draw an “ideal woman” and “ideal man” and identify characteristics considered inside or outside the community’s “gender box.” Facilitators encourage participants to examine who benefits from these expectations, how departures from them are socially sanctioned, and how gendered expectations affect reproductive autonomy. Facilitators should avoid reinforcing stereotypes and emphasize that the activity examines social expectations rather than inherent characteristics of women or men
Things People Say	60 min	Participants identify stigmatizing words and labels directed toward adolescents, girls who engage in sexual activity, and girls who use contraception; examine the effects of stigmatizing language; and practice replacing judgmental language with respectful alternatives	Labeling and stereotyping; stigma marking; moral judgment; social norms; language as a mechanism of stigma and power	Participants brainstorm words, phrases, slang, and community expressions used to describe the identified groups. Facilitators guide discussion of how labels affect self-perception, social relationships, and willingness to seek SRH information or services. Facilitators should establish expectations for respectful participation and clarify that stigmatizing terms are being examined critically rather than endorsed
Stigma Reflection	60 min	Participants recognize the emotional and social effects of exclusion and judgment; relate personal experiences of being treated as different to broader processes of stigma; and demonstrate empathy toward people experiencing contraceptive or SRH stigma	Experienced and anticipated stigma; stigma manifestations; empathy; perspective-taking; critical consciousness	Participants privately reflect on a time when they felt isolated, rejected, or judged for being perceived as different. Sharing personal experiences is voluntary. Facilitators guide reflection on emotional and social consequences and connect these experiences with contraceptive and SRH stigma. Facilitators should monitor discomfort, avoid pressuring disclosure, and remind participants that they may decline to share
Challenge the Stigma, Be the Change	60 min	Participants identify opportunities to respond to everyday stigma; practice assertive and respectful communication; and develop individual and collective strategies for challenging stigmatizing comments and treatment	Agency and empowerment; stigma resistance; collective action; communication self-efficacy; disruption of stigma manifestations	Participants respond to role-play scenarios involving stigmatizing language or treatment in peer, family, community, or service settings. Facilitators support participants in practicing safe, context-sensitive responses and discussing when direct confrontation may not be appropriate. The activity concludes by identifying realistic actions participants can take individually or with peer and adult support

*Note*. Activities were adapted from the London School of Hygiene and Tropical Medicine’s Expanded Total Facility Approach stigma-reduction curriculum for use with adolescent girls and young women in community settings. SRH = Sexual and Reproductive Health.

Before intervention delivery, the four facilitators completed a 1-day training led by the principal investigator. Training addressed the conceptualization of stigma, adolescent contraceptive and SRH stigma, gender norms, participatory facilitation, delivery of each workshop activity, management of sensitive discussions, research ethics, confidentiality, and procedures for responding to participant distress or safeguarding concerns. Facilitators reviewed the structured workshop guide and discussed the content, sequencing, and facilitation of each activity. Facilitator competency was not formally assessed. Consistency of delivery was supported through use of the structured facilitator guide. Attendance was documented at each session, and facilitators debriefed with the principal investigator after each session to review activity delivery, participant engagement, implementation challenges, and any adjustments required for subsequent sessions. A standardized fidelity rating instrument was not used in this initial pilot.

### Data Collection

#### Quantitative Measures

Participants completed self-administered surveys immediately before and after the workshops to examine preliminary pre–post changes. The selected measures had been developed or evaluated in other African or cross-cultural settings but had not previously been fully validated among Tanzanian adolescents. Accordingly, their use in this pilot was exploratory.

- Contraceptive Use Stigma (CUS): This measure assesses stigmatizing attitudes and beliefs related to contraceptive use and was previously used in Kenya (Meurice et al., 2021).
- Adolescent SRH Stigma: This scale assesses stigma related to sexual activity, pregnancy, modern contraception, and abortion and was originally developed and validated among young women in Ghana (Hall et al., 2018; Logie et al., 2019). It has subsequently been used with adolescent and young adult populations in other sub-Saharan African settings (Logie et al., 2019).
- Sexual Double Standards: This measure assesses gendered expectations concerning sexual behavior and relationships and was developed through the cross-cultural Global Early Adolescent Study (Moreau et al., 2019, 2021).

Survey instruments were administered in Kiswahili. Survey instruments were translated from English into Kiswahili and reviewed collaboratively by bilingual members of the Tanzanian research team for linguistic clarity, cultural relevance, and conceptual consistency with the original measures. Because the scales had not been fully validated for this specific population and context, internal consistency was assessed in the study sample, and findings were interpreted in conjunction with participant and facilitator feedback concerning item comprehension.

Surveys were self-administered, with trained research staff available to clarify the meaning of instructions or individual items without suggesting a response. This support was provided particularly for younger participants and those with limited prior exposure to survey-based research.

#### Qualitative Data

Qualitative data were collected through three audio-recorded focus group discussions with participants and facilitators 3 weeks following completion of the workshops: two 60- to 90-min focus group discussions with participants (one urban group, one rural group) and one 30-min focus group discussion with facilitators. Participant discussions explored experiences of participation, perceived relevance and acceptability, reflections on stigma and gender norms, and suggestions for improvement. Facilitator discussions additionally addressed implementation feasibility, challenges, and observations of participant engagement.

### Data Analysis

Quantitative analyses examined preliminary pre–post changes in stigma-related outcomes and explored differences by setting and participant characteristics. Descriptive statistics were used to summarize participant characteristics and baseline measures. Paired-samples *t*-tests assessed within-participant changes in each outcome, while independent-samples *t*-tests and chi-square tests were used to examine baseline differences by selected sociodemographic characteristics. Analyses by urban and rural setting were conducted exploratorily.

Because this pilot was designed primarily to assess feasibility and acceptability and included a small sample without a control group, the outcome analyses were not intended to provide definitive tests of intervention efficacy. Interpretation therefore emphasized the direction and magnitude of observed changes using mean differences and Cohen’s *d* effect sizes, together with 95% confidence intervals, rather than relying solely on statistical significance. Effect sizes are less directly dependent on sample size than *p-*values and can provide preliminary information to inform the design and sample-size assumptions of a future controlled evaluation. Nevertheless, effect estimates from this small pilot are imprecise and should not be interpreted as evidence of effectiveness. All analyses were conducted using IBM SPSS Statistics version 29.0.2.0, with two-sided statistical significance set at *p* < .05.

Qualitative data were analyzed using reflexive thematic analysis following Braun and Clarke’s approach, which recognizes that themes are actively constructed through researchers’ interpretive engagement with the data (Braun & Clarke, 2022). The research team included members with expertise in adolescent SRH, stigma research, and community-based programming in Tanzania, and reflexive discussions were used throughout analysis to consider how positionality and disciplinary perspectives shaped interpretation.

An initial hybrid deductive-inductive coding framework was developed drawing on stigma theory and participants’ narratives (DeCuir-Gunby et al., 2010). Two researchers independently coded the data and met iteratively to refine codes and construct themes through consensus and reflexive discussion. Themes were developed through sustained engagement with the data to capture shared meanings related to stigma, gender norms, and workshop experiences.

Quantitative and qualitative data were initially analyzed separately and subsequently integrated through a side-by-side comparison of findings. Integration focused on convergence, complementarity, and divergence across the two data sources. Quantitative changes in CUS, broader adolescent SRH stigma, and sexual double standards were compared with qualitative themes concerning contraceptive attitudes, gender norms, community and institutional barriers, confidence, and participant experiences. A mixed-methods joint display was developed to summarize these relationships and identify how qualitative findings supported, expanded, or differed from the quantitative results. The integrated interpretation was used to distinguish outcomes that appeared responsive to the brief workshops from broader norms and structural barriers that remained unchanged or required more sustained intervention.

### Ethical Considerations

Ethical approval was obtained from Queen’s University Health Sciences and Research Ethics Board in Canada (No. 6045690) and Kilimanjaro Christian Medical Centre (No. 2844) in Tanzania. Written informed consent was obtained directly from all participants, including separate permission for the use of participant-created artwork. In accordance with the approvals granted by the Canadian and Tanzanian research ethics authorities, participants younger than 18 years were permitted to provide their own informed consent without parental or guardian permission, consistent with ethical guidance concerning adolescent participation in SRH research and recognition of adolescents’ evolving capacity to make informed decisions regarding their own SRH (Society for Adolescent Health & Medicine, 2025). Requiring parental permission could have excluded adolescents who were particularly affected by SRH stigma or who were unable or unwilling to disclose their interest in SRH topics to a parent or guardian (Society for Adolescent Health & Medicine, 2025). The consent process emphasized voluntariness, confidentiality, the right to decline individual activities or questions, and the right to withdraw without penalty. Confidentiality was ensured using de-identified transcripts, unique participant IDs, secure data storage procedures, and restricted access to study files. Participants received transportation reimbursement and meals; no other incentives were provided.

## Results

### Participant Characteristics

The sample included 40 AGYW: 20 from the urban setting and 20 from the rural setting. Participants had a mean age of 18.38 years (*SD* = 1.43). Most were single (80.00%), had completed secondary education (70.00%), had never been pregnant (80.00%), and had never used contraception (87.50%). At baseline, 62.50% were categorized as having good contraceptive knowledge and 80.00% as having good general SRH knowledge; however, 77.50% believed that contraception could cause harmful side effects. Rural participants generally demonstrated less familiarity with contraceptive methods and SRH terminology than urban participants and had significantly higher baseline CUS scores (*p* = .019). Selected participant characteristics are presented in Table 2.

**Table 2.** Sociodemographic Characteristics and Baseline Knowledge of Participants in Urban and Rural Kilimanjaro, Tanzania, February 2025 (*N* = 40).

Characteristic	*n* (%)
Age, years, mean (*SD*)	18.38 (1.43)
Setting	
Urban	20 (50.00)
Rural	20 (50.00)
Relationship status	
Single	32 (80.00)
In a relationship	8 (20.00)
Highest level of education	
Primary	8 (20.00)
Secondary	28 (70.00)
Certificate or college	4 (10.00)
Ever been pregnant	
No	32 (80.00)
Yes	7 (17.50)
Unsure	1 (2.50)
Ever used contraception	
No	35 (87.50)
Yes	5 (12.50)
Contraceptive knowledge	
Poor	15 (37.50)
Good	25 (62.50)
General SRH knowledge	
Poor	8 (20.00)
Good	32 (80.00)
Believed contraception could cause harmful side effects	
No	9 (22.50)
Yes	31 (77.50)

*Note*. SRH = Sexual and Reproductive Health.

### Quantitative Findings

Mean CUS scores decreased from 2.34 (*SD* = 0.86) before the workshops to 1.75 (*SD* = 0.71) after the workshops (*p* < .001, Cohen’s *d* = −0.76; Figure 1A). No statistically significant pre–post differences were observed for the adolescent SRH stigma scale, although score distributions showed a shift toward lower values following the workshops.

**Figure 1.**
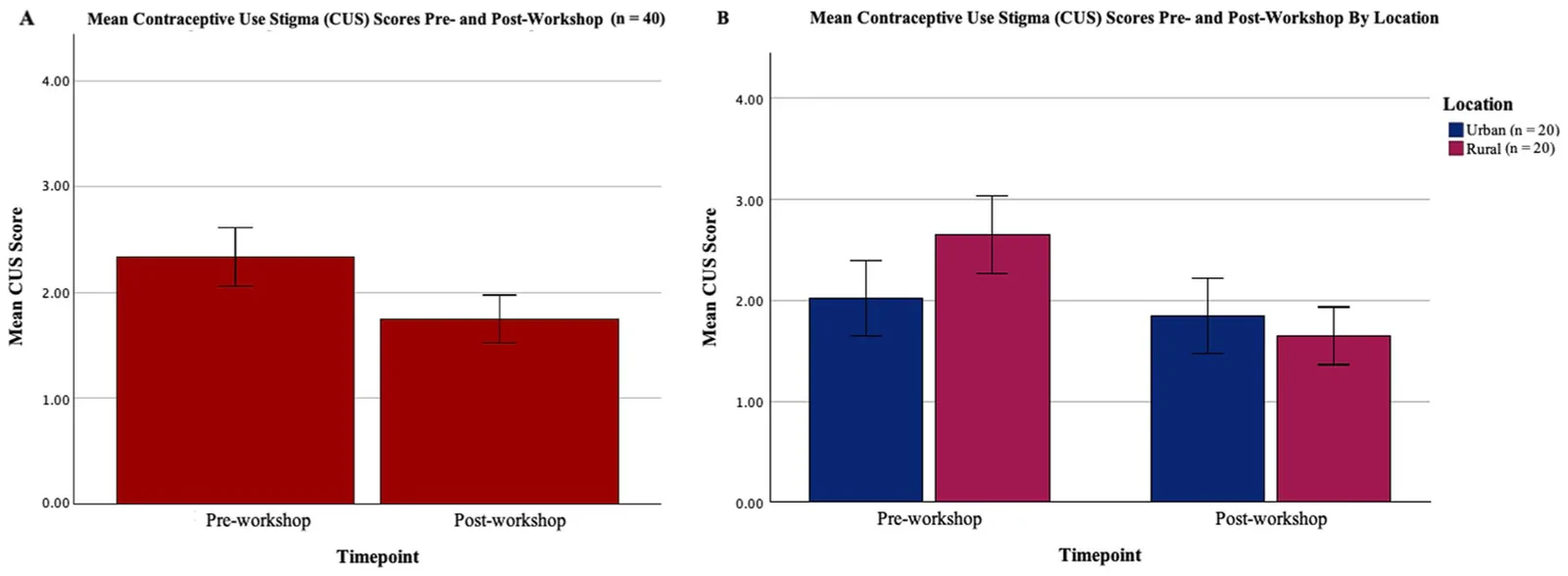
Changes in contraceptive use stigma (CUS) scores. (A) Mean CUS scores before and immediately after the workshops for the full sample (*n* = 40). CUS scores decreased from pre- to post-workshop (*p* < .001; Cohen’s *d* = −0.76). (B) Mean CUS scores by setting before and immediately after the workshops (urban, *n* = 20; rural, *n* = 20). *Note*. Rural participants had significantly higher baseline CUS scores than urban participants (*p* = .02); this difference was not statistically significant after the workshops. Error bars represent 95% confidence intervals. Higher scores indicate greater contraceptive use stigma.

Internal consistency was acceptable for the adolescent SRH stigma scale at pre-workshop (Cronbach’s *a* = .82) and post-workshop (*a* = .72) and for the CUS scale at pre-workshop (*a* = .81) and post-workshop (*a* = .78). Internal consistency for the sexual double standards scale was below the conventional threshold of .70 at both pre-workshop (*a* = .70) and post-workshop (*a* = .68), limiting interpretation of the total score. Item-level analyses and post hoc item removal were not undertaken because they were not prespecified and the small pilot sample was insufficient to support data-driven modification of the scale. No statistically significant pre–post difference was observed for the sexual double standards scale; however, this finding should be interpreted cautiously given its lower internal consistency.

At baseline, rural participants reported significantly higher CUS scores than urban participants (*p* = .02). This difference was no longer statistically significant at post-intervention (Figure 1B).

### Qualitative Findings

Six qualitative themes were developed concerning participants’ contraceptive attitudes, gender norms, community and institutional barriers, confidence and peer support, workshop acceptability, and recommendations for implementation. Table 3 presents an integrated summary of these themes, related quantitative findings, and implications for practice.

**Table 3.** Mixed-Methods Integration of Findings From Participatory Stigma-Reduction Workshops in Urban and Rural Kilimanjaro, Tanzania, February to March 2025.

Qualitative theme	Interpretive summary	Representative quotation	Corresponding quantitative finding	Implication for practice
Knowledge and Shifts in Contraceptive Attitudes	Participants demonstrated awareness of the potential benefits of contraception but described their attitudes and decisions as shaped by concerns about side effects, perceived readiness, and moral judgments. Some participants reported becoming less judgmental toward adolescent contraceptive use following the workshops	“I used to think people who use birth control are bad, but I’ve realized that those who use birth control are just trying to reach their goals.” (Rural, P-22)	CUS scores decreased significantly from pre- to post-workshop (*p* < .001, Cohen’s *d* = −0.76), supporting qualitative accounts of reduced moral judgment toward contraceptive use	Participatory activities should address both contraceptive information and the moral narratives surrounding adolescent use. Practical, age-appropriate information about contraceptive methods and side effects may further support informed decision-making
Gender Norms and Sexual Double Standards	Participants identified restrictive expectations of femininity and greater social freedom for boys. They described contraceptive use by girls as challenging gendered expectations concerning sexual restraint and male authority	“When a woman uses family planning, she is seen as having taken over the man’s authority to decide on matters of sexuality.” (Urban, P-20)	No statistically significant change was detected on the sexual double standards measure. Its lower internal consistency also limited interpretation. The qualitative findings nevertheless demonstrated that participants could identify and critically discuss gendered double standards	Brief workshops may initiate reflection but may be insufficient to change deeply embedded gender norms. Gender-transformative content should be reinforced over time and should engage boys, families, and community actors in addition to adolescent girls
Community and Institutional Barriers	Participants described stigma as reinforced by families, community members, religious discourse, pharmacies, and healthcare providers. Anticipated judgment, breaches of confidentiality, and age-based scolding discouraged access to SRH services	“When she goes to seek family planning services, she finds the doctor scolding her, telling her that she is still too young.” (Rural, P-26)	Broader adolescent SRH stigma did not change significantly following the workshops. The qualitative findings suggest that persistent community- and institutional-level barriers may help explain the absence of measurable change in broader stigma	Adolescent workshops should be accompanied by interventions for healthcare providers, parents, schools, religious leaders, and other community influencers. Provider-focused programming should emphasize confidentiality, nonjudgmental communication, and adolescent-responsive care
Confidence, Agency, and Peer Support	Participants described greater comfort discussing SRH with peers and adults and greater confidence in sharing information and challenging stigmatizing judgments. These perceived changes extended beyond the attitudinal outcomes measured quantitatively	“I feel free to talk in front of my elders, my parents . . . family planning benefits all age groups, both male and female.” (Urban, P-04)	Communication confidence and agency were not measured quantitatively in this pilot. Qualitative findings therefore expanded the quantitative results by identifying potential outcomes for inclusion in future evaluations	Future programs should incorporate opportunities to practice communication, peer support, and responses to stigmatizing statements. Future evaluations should measure communication confidence, self-efficacy, agency, and peer support behaviors
Acceptability, Safety, and Participant Engagement	Participants and facilitators described the workshops as relevant and engaging. Trust and participation increased as the sessions progressed, and activities directly connected to participants’ experiences generated particularly strong engagement	“Even during that topic of things people say, the participation was very good, because it was touching them directly.” (Facilitator, F-01)	All 40 participants completed the pre- and post-workshop surveys, and attendance was high across sessions. These findings converged with qualitative accounts of acceptability and engagement	Programs should use trained local facilitators, participatory activities, and clear group expectations concerning respect, confidentiality, and voluntary disclosure. Attendance and engagement should continue to be monitored alongside participant-reported acceptability
Implementation Recommendations	Participants and facilitators recommended additional visual and practical contraceptive content, mental health support, and extension of programming beyond adolescent participants. These recommendations identified areas for refining the intervention before a larger evaluation	“Urban [girls], they were so informative . . . but to rural [girls] we struggled.” (Facilitator, F-01)	Rural participants had higher baseline CUS scores than urban participants (*p* = .02), and facilitators reported differences in baseline familiarity with SRH concepts. The urban–rural CUS difference was no longer statistically significant after the workshops	Intervention delivery should be responsive to differences in baseline knowledge and local context. Future adaptations should include practical and visual materials, integrated mental health content, and tailored facilitation support for groups requiring additional clarification

*Note*. Quantitative findings were interpreted as exploratory because this pilot included a small sample and no control group. CUS = Contraceptive Use Stigma; SRH = Sexual and Reproductive Health.

#### Knowledge and Shifts in Contraceptive Attitudes

This theme illustrates that participants’ contraceptive attitudes reflected an interaction between knowledge, perceived benefits, concerns about side effects, personal readiness, and moral judgments. Although participants often recognized contraception as supporting health and future goals, their willingness to use or endorse it remained context-dependent. Some adolescents articulated holistic conceptions of SRH that encompassed physical, mental, and social wellbeing. As one rural participant explained,

Sexual health entails engaging in safe, consensual, and pleasurable sexual practices, while reproductive health refers to the state of having safe sexual activities that are physically, mentally, and socially sound. (Rural, P-37)

Many participants recognized contraception as a tool for protecting future opportunities and supporting educational and personal goals, while simultaneously expressing concern about side effects or misuse. An urban participant noted,

The methods of contraception primarily reduce deaths, decrease adolescent pregnancies, and help individuals achieve their goals. However, if these modern methods are misused, they can lead to adverse effects later on. (Urban, P-19)

Despite this awareness, decisions about contraceptive use were often described as deeply contextual rather than purely informational. Some adolescents expressed hesitation based on feelings of readiness or comfort, while others framed contraceptive use as a pragmatic response to risk. One participant stated,

For me, I do not feel comfortable using those pills [oral contraception] right now. (Urban, P-01)

In contrast, another explained,

I feel good, and I think it is perfectly fine . . . perhaps I have my own goals and maybe I am in a risky situation I can use [contraception]. (Urban, P-13)

Together, these narratives highlight how adolescents described navigating contraceptive decision-making within contexts shaped by stigma and moral surveillance.

#### Gender Norms and Sexual Double Standards

This theme demonstrates how contraceptive and SRH stigma was embedded within gendered expectations that emphasized sexual restraint, obedience, and respectability for girls while permitting greater sexual freedom and authority for boys. Through the “Outside the Gender Box” activity, adolescents articulated rigid ideals of femininity and masculinity that structured acceptable behavior. The “ideal woman” was portrayed as modest, obedient, and sexually pure, while the “ideal man” was characterized as dominant, emotionally restrained, and financially capable (Figure 2). These constructions positioned girls’ sexuality as something to be controlled and monitored, while granting boys greater social liberty.

**Figure 2.**
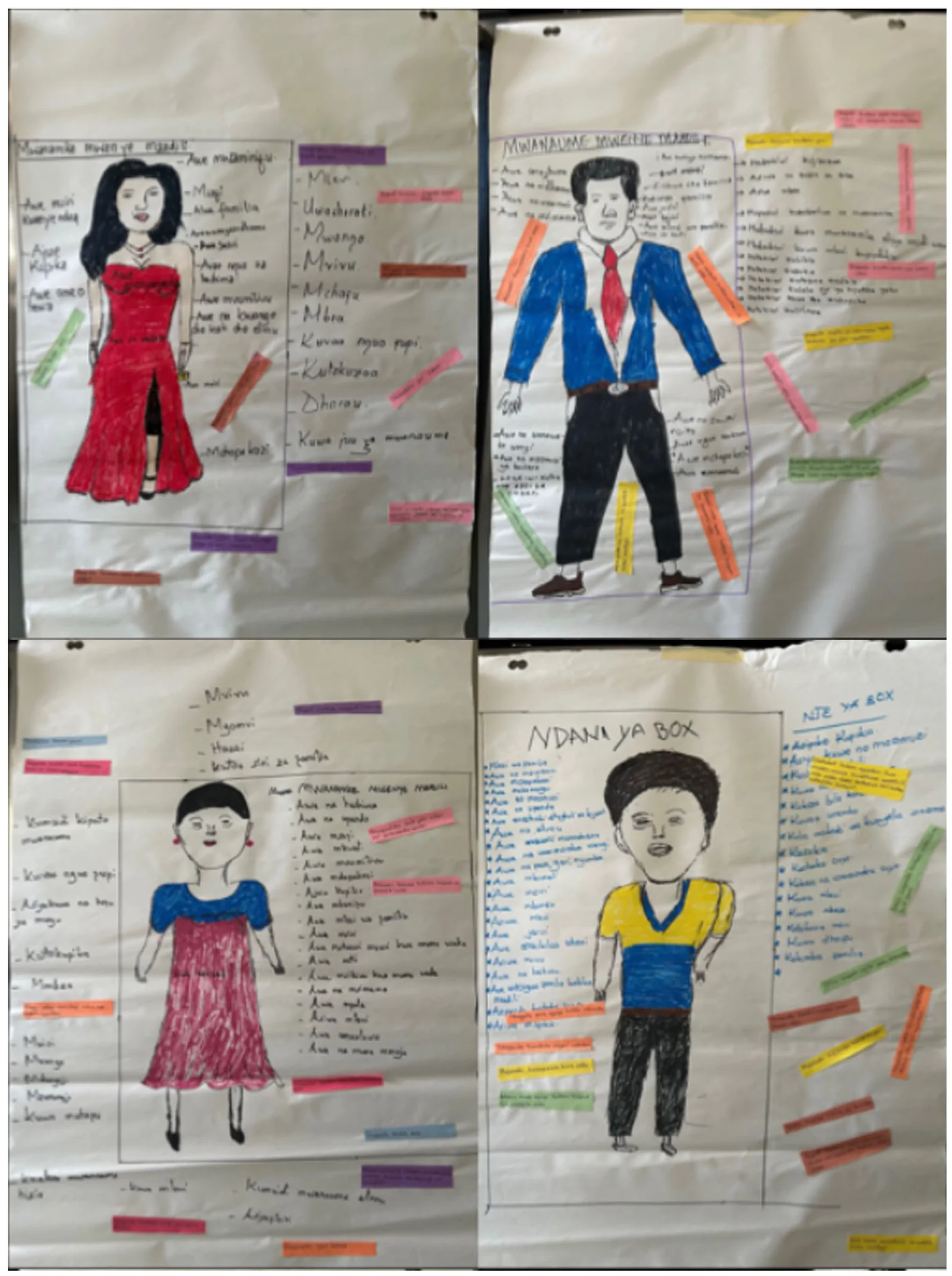
Participant drawings from the outside the gender box activity. *Note*. Urban participant drawings are displayed in the top row, with an “ideal woman” on the left and the “ideal man” on the right. Rural participant drawings are displayed in the bottom row, with the “ideal woman” on the left and the “ideal man” on the right. Across the drawings and accompanying discussions, the “ideal woman” was associated with modesty, obedience, domestic responsibility, and sexual restraint, whereas the “ideal man” was associated with authority, financial provision, emotional restraint, and greater sexual freedom. These annotations summarize the gender expectations identified by participants during the activity and do not indicate endorsement by the participants or research team.

Participants described experiencing stigma or punishment for behaviors perceived as deviating from feminine norms, including clothing choices, social interactions with boys, and aspirations related to education or employment. A rural participant captured the prevailing double standard succinctly:

For boy’s adolescent, engaging in sexual activities appears to be a customary practice; however, for a girl, frequent sexual behaviour is perceived as prostitute. (Rural, P-30)

Contraceptive use was often framed as a moral transgression that threatened male authority and gendered power relations. As one urban participant explained,

When a woman uses family planning, she is seen as having taken over the man’s authority to decide on matters of sexuality. (Urban, P-20)

Participants described contraceptive use as socially risky within gendered expectations that emphasized control over girls’ sexuality.

#### Community and Institutional Barriers

This theme shows that participants understood stigma as extending beyond individual attitudes and being reproduced through family relationships, community expectations, religious discourse, and health care interactions. Community attitudes toward contraception were widely perceived as negative and shaped by misinformation and moral judgment. An urban adolescent explained,

The prevailing perspective in the community is a negative one regarding the use of family planning . . . they do not understand how to effectively utilize it . . . therefore, they experience more negative outcomes. (Urban, P-20)

Religious discourse was frequently cited as reinforcing stigma, even as participants critically reflected on the material consequences of large family size. One participant noted,

They claim [in the Quran] that using family planning is a sin . . . however, when there are many children, they often lack proper upbringing, shelter, and even food. (Urban, P-01)

Barriers were particularly pronounced within health care settings. Participants reported fear of gossip, breaches of confidentiality, and age-based scolding by providers. One adolescent shared,

You might go to a pharmacy and get good service, but when you’re treated, they’ll tell others . . . it’ll turn into rumors like you’re a promiscuous person. (Urban, P-17)

Rural participants emphasized similar experiences, particularly being dismissed as too young to seek services:

When she goes to seek family planning services, she finds the doctor scolding her, telling her that she is still too young. (Rural, P-26)

These narratives illustrate how stigma persists even when services are nominally available, discouraging adolescents from accessing information and care.

#### Confidence, Agency, and Peer Support

This theme captures participants’ perceptions that the workshops strengthened their confidence to discuss SRH, communicate with peers and adults, and respond more empathetically to contraceptive use, even though broader community barriers remained. One urban participant shared,

I feel free to talk in front of my elders, my parents . . . family planning benefits all age groups, both male and female. (Urban, P-04)

Several adolescents described changes in moral reasoning around contraception, moving from judgment to empathy. A rural participant reflected,

I used to think people who use birth control are bad, but I’ve realized that those who use birth control are just trying to reach their goals. (Rural, P-22)

#### Acceptability, Safety, and Participant Engagement

This theme indicates that the participatory format was acceptable and engaging, particularly once trust was established and activities connected directly with participants’ experiences of stigma. Facilitators noted differences in baseline knowledge, with urban participants demonstrating greater familiarity with SRH concepts, while rural groups initially required more clarification. As one facilitator explained,

Urban [girls] they were so informative . . . but to rural [girls] we struggled (F-01)

Interactive activities were widely viewed as effective in fostering engagement, particularly those that addressed stigma directly. One facilitator noted,

Even during that topic of things people say, the participation was very good, because it was touching them directly. (F-01)

Participants described the workshops as spaces where they felt safe to share experiences and learn from peers, reinforcing the acceptability of the participatory format.

#### Implementation Recommendations

This theme identifies participants’ and facilitators’ priorities for strengthening the intervention, including more practical and visual contraceptive content, attention to emotional wellbeing, and engagement beyond the adolescent workshop groups. Facilitators emphasized the emotional burden many adolescents carried and recommended integrating mental health content. Participants called for more practical and visual learning approaches, including demonstrations of contraceptive methods. Finally, both participants and facilitators stressed the importance of extending education beyond adolescents themselves, to foster community involvement.

Collectively, these reflections highlight opportunities for refining and scaling the intervention through integrated content, emotional wellbeing, and broader community engagement.

## Discussion

This mixed-methods pilot provides preliminary evidence that participatory stigma-reduction workshops can be feasibly and acceptably delivered to AGYW in community-based settings in Tanzania. Complete pre- and post-workshop data collection, high attendance, and positive feedback from participants and facilitators support the practicality of delivering stigma-focused programming through a trusted local organization. However, because the study did not include a control group or longer-term outcome assessment, the quantitative findings should be interpreted as preliminary and should not be attributed definitively to the intervention.

CUS scores decreased from pre- to post-workshop, with a moderate-to-large standardized effect size. This finding suggests that contraceptive-specific judgments may be responsive to participatory activities that directly identify and challenge stigmatizing language, assumptions, and moral narratives. The qualitative findings supported this interpretation: participants described becoming less judgmental toward contraceptive use and more likely to understand it as a strategy for protecting health and achieving educational or personal goals. Nevertheless, the effect estimate is imprecise because of the small sample, and regression to the mean, social desirability, repeated testing, or other influences cannot be excluded.

In contrast, broader adolescent SRH stigma and sexual double standards did not change significantly. These outcomes may represent more diffuse and deeply embedded systems of gendered expectations concerning sexuality, respectability, obedience, and male authority. Such norms are reinforced across families, peer groups, religious institutions, communities, and health care settings and may therefore be less responsive to a brief intervention delivered only to AGYW. The workshops may have facilitated critical recognition of these norms without producing an immediate measurable change in participants’ responses. Interpretation of the sexual double standards result is further limited by the scale’s lower internal consistency, which may indicate that the measure did not adequately capture locally meaningful manifestations of sexual double standards. Longer, repeated, and explicitly gender-transformative programming may be required to influence these broader norms.

Rural participants had significantly higher baseline CUS scores than urban participants, although this difference was no longer statistically significant following the workshops. This baseline difference may reflect variation in exposure to SRH information, geographic access to adolescent-responsive services, and the social visibility of contraceptive use in more geographically dispersed communities. Facilitators similarly observed that rural participants initially required more clarification of SRH concepts and terminology. However, these explanations remain tentative because the pilot was not powered to examine setting-specific effects, and the disappearance of statistical significance after the workshops does not establish that the intervention eliminated the urban-rural difference. Future studies should examine how service access, community norms, educational exposure, and implementation conditions influence stigma and intervention responsiveness across settings.

Qualitative findings provided important context for interpreting these quantitative results. Participants described greater comfort discussing SRH, increased confidence communicating with peers and adults, and greater empathy toward adolescents who use contraception. At the same time, accounts of provider judgment, parental control, breaches of confidentiality, religious disapproval, and community surveillance remained prominent. These findings reinforce the Health Stigma and Discrimination Framework’s conceptualization of stigma as a multilevel process and the Stigma Power Process Model’s emphasis on stigma as a mechanism that maintains gendered social control. The findings suggest that adolescent-focused workshops may initiate reflection and changes in individual attitudes but cannot independently alter the interpersonal, institutional, and structural conditions through which stigma is reproduced.

Multilevel intervention strategies are therefore needed. Adolescent-focused workshops could be combined with parent and caregiver dialogue, gender-transformative programming for boys and young men, provider training in confidential and non-judgmental adolescent-responsive care, and school-based efforts to address stigmatizing language and exclusion. Engagement with religious leaders, community leaders, and other local influencers may help identify culturally appropriate ways to challenge moral judgment while supporting adolescent health and wellbeing. At the policy and health-system levels, stigma-reduction programming should be accompanied by clear confidentiality procedures, supportive supervision, accessible youth-responsive services, and mechanisms through which adolescents can safely report stigmatizing treatment.

Because quantitative outcomes were assessed immediately after the workshops, it is unknown whether the observed reduction in contraceptive stigma persisted over time or translated into changes in communication, care-seeking, contraceptive decision-making, or supportive behavior. Although the follow-up focus groups conducted 3 weeks after the workshops provided short-term insight into participants’ experiences, they cannot establish sustained attitudinal or behavioral change. Future controlled studies should include longer-term assessments and examine potential mechanisms, including critical reflection, communication confidence, perceived peer norms, and reproductive agency.

The study also has several strengths. Its mixed-methods design allowed preliminary quantitative changes to be interpreted alongside participants’ and facilitators’ accounts of acceptability, engagement, and perceived effects. Implementation in both urban and rural settings provided early insight into contextual variation, while collaboration with a Tanzanian community-based organization supported linguistic, cultural, and practical relevance. The use of trained local female facilitators and participatory activities helped create opportunities for dialogue about sensitive and socially regulated topics. Finally, the study’s explicit theoretical grounding supported interpretation of both individual-level attitudinal changes and the persistence of broader gendered and structural barriers.

This study has limitations. The small, non-probability sample and absence of a control group limited statistical power and precluded causal attribution of the observed changes to the intervention. Quantitative outcomes were assessed immediately after the workshops, and the focus groups were conducted 3 weeks later; therefore, the study could not determine whether perceived or measured changes persisted or influenced subsequent communication, care-seeking, or contraceptive decision-making. Reliance on self-reported outcomes may have introduced social desirability and Hawthorne effects, particularly because participants were aware that the workshops sought to challenge stigma. Responses during focus group discussions may also have been influenced by group dynamics, perceived facilitator or researcher expectations, and reluctance to express dissenting views. Although survey support was intended to facilitate comprehension, interaction with research staff may have influenced some responses. The lower internal consistency of the sexual double standards scale and the absence of full validation of the measures among Tanzanian adolescents further limited interpretation of the quantitative findings. Facilitator competency and intervention fidelity were not formally assessed; although attendance records and post-session debriefings with the principal investigator supported implementation monitoring, these procedures did not permit standardized assessment of delivery across facilitators and settings. The study was conducted in two communities in the Kilimanjaro Region, limiting generalizability to adolescents in other geographic, social, and service contexts. Finally, the small urban and rural groups did not support robust examination of contextual differences in implementation or outcomes. Accordingly, the findings should be interpreted as exploratory and hypothesis-generating and require confirmation through larger, controlled studies with longer-term follow-up and more comprehensive implementation assessment.

## Implications for Practice

The findings identify several actions for health promotion practitioners working in adolescent SRH. Community-based health promoters and nongovernmental organizations should incorporate explicit stigma-reduction activities into adolescent SRH programming rather than relying primarily on information provision. Facilitated discussion, critical reflection, visual activities, and role-play can help adolescents identify stigmatizing language, examine gendered expectations, and practice responding to judgment. Programs should establish clear expectations regarding respect, confidentiality, voluntary disclosure, and participants’ right to decline sensitive activities.

SRH educators and program designers should distinguish contraceptive-specific stigma from broader SRH stigma and sexual double standards. Contraceptive-specific attitudes may respond to brief, targeted activities, whereas deeply embedded gender norms likely require repeated and explicitly gender-transformative programming. Materials should use locally familiar language and scenarios and include practical, visual information about contraceptive methods and potential side effects. Content should be adapted to participants’ baseline knowledge rather than delivered uniformly across settings.

Health care providers, pharmacists, and health-service managers should examine how judgmental communication, age-based scolding, and breaches or perceived breaches of confidentiality discourage adolescents from seeking care. Training should emphasize nonjudgmental communication, confidentiality, informed contraceptive choice, and respectful care regardless of age or relationship status. Health-service managers should establish clear confidentiality procedures, provide supportive supervision, and create safe mechanisms through which adolescents can report stigmatizing treatment.

School-based health promoters, teachers, and youth-serving professionals should address the language and gendered expectations through which contraceptive and SRH stigma is reinforced. Programming should provide opportunities for adolescents to practice communication and peer support skills and should include appropriate referral pathways to confidential SRH and mental health services. Schools should avoid disciplinary or educational practices that shame, exclude, or disclose the SRH concerns of adolescent students.

Practitioners responsible for family and community engagement should complement adolescent-focused programming with dialogue involving parents and caregivers, boys and young men, religious leaders, and other community influencers. Engagement should focus on how stigma affects adolescent health and service access while identifying locally acceptable ways to support confidentiality, reproductive autonomy, and respectful communication. Because adolescents alone cannot change the interpersonal and institutional environments that sustain stigma, responsibility for change should be shared across families, communities, schools, and health systems.

Across these roles, practitioners should monitor more than attendance and knowledge acquisition. Implementation assessment should include participant-reported acceptability and safety, engagement, facilitator delivery, experiences of stigma, and variation across urban and rural settings. These data can guide iterative adaptation before broader implementation.

## Implications for Policy

At a policy level, findings support the integration of stigma-reduction components within youth-friendly SRH strategies, particularly those implemented through community-based platforms. Policies that promote nonjudgmental, confidential, and adolescent-centered care may be strengthened by incorporating participatory approaches that explicitly address the social and moral norms shaping adolescents’ access to services. Embedding stigma-reduction principles within existing adolescent health policies and program guidelines may help address persistent barriers to care that are not fully resolved through service availability alone.

## Implications for Research

From a research perspective, further evaluation using larger and more rigorously controlled designs is needed to assess the sustainability of stigma-related changes and potential impacts on care-seeking behaviors and supportive social environments. Future studies should prioritize the refinement and validation of stigma measurement tools for adolescent populations, with attention to linguistic clarity, cultural relevance, and contextual sensitivity. Additional research exploring multilevel intervention strategies, including family and community engagement, will be important for strengthening the evidence base for stigma-focused health promotion interventions and advancing adolescent SRH equity.
